# Neurofilament light chain associates with IVH and ROP in extremely preterm infants

**DOI:** 10.1038/s41390-024-03587-5

**Published:** 2024-09-24

**Authors:** Ulrika Sjöbom, Annika Öhrfelt, Aldina Pivodic, Anders K. Nilsson, Kaj Blennow, Henrik Zetterberg, William Hellström, Hanna Danielsson, Lotta Gränse, Karin Sävman, Dirk Wackernagel, Ingrid Hansen-Pupp, David Ley, Ann Hellström, Chatarina Löfqvist

**Affiliations:** 1 Learning and Leadership for Health Care Professionals, Institute of Health and Care Science at Sahlgrenska Academy, University of Gothenburg, Gothenburg, Sweden; 2 Department of Clinical Neuroscience, Institute of Neuroscience and Physiology, Sahlgrenska Academy, University of Gothenburg, Gothenburg, Sweden; 3 Department of Psychiatry and Neurochemistry, Institute of Neuroscience and Physiology, The Sahlgrenska Academy at the University of Gothenburg, Mölndal, Sweden; 4 Clinical Neurochemistry Laboratory, Sahlgrenska University Hospital, Mölndal, Sweden; 5 Paris Brain Institute, ICM, Pitié-Salpêtrière Hospital, Sorbonne University, Paris, France; 6 Neurodegenerative Disorder Research Center, Division of Life Sciences and Medicine, and Department of Neurology, Institute on Aging and Brain Disorders, University of Science and Technology of China and First Affiliated Hospital of USTC, Hefei, PR China; 7 Department of Neurodegenerative Disease, University College of London Institute of Neurology, London, UK; 8 UK Dementia Research Institute, University College of London, London, UK; 9 Hong Kong Center for Neurodegenerative Diseases, Clear Water Bay, Hong Kong, China; 10 Wisconsin Alzheimer’s Disease Research Center, University of Wisconsin School of Medicine and Public Health, University of Wisconsin-Madison, Madison, WI USA; 11 Department of Pediatrics, Institute of Clinical Sciences, Sahlgrenska Academy, University of Gothenburg, Gothenburg, Sweden; 12 Centre for Translational Microbiome Research, Department of Microbiology, Tumor and Cell Biology, Karolinska Institutet, Stockholm, Sweden; 13 Sach’s Children’s and Youth Hospital, Södersjukhuset, Stockholm Sweden; 14 Department of Clinical Sciences, Ophthalmology, Lund University, Skåne University Hospital, Lund, Sweden; 15 Region Västra Götaland, Department of Neonatology, The Queen Silvia Children’s Hospital, Sahlgrenska University Hospital, Gothenburg, Sweden; 16 Department of Clinical Science, Intervention and Technology (CLINTEC), Karolinska Institutet, Stockholm, Sweden; 17 Division of Neonatology, Department of Pediatrics, University Medical Center of the Johannes Gutenberg-University Mainz, Mainz, Germany; 18 Department of Clinical Sciences Lund, Pediatrics, Lund University, Skåne University Hospital, Lund, Sweden

## Abstract

**Background:**

Neurofilament light chain (NfL) is known for indicating adult brain injury, but the role of NfL in extremely preterm infants is less studied. This study examines the relationship between NfL and neurovascular morbidities in these infants.

**Methods:**

A secondary analysis of the Mega Donna Mega trial was conducted on preterm infants <28 weeks gestational age (GA). The study measured NfL levels and proteomic profiles related to the blood-brain barrier in serum from birth to term-equivalent age, investigating the association of NfL with GA, retinopathy of prematurity (ROP), intraventricular hemorrhage (IVH), and blood-brain barrier proteins.

**Results:**

Higher NfL levels were seen in the first month in infants with severe IVH and for those born <25 weeks GA (independent of ROP or IVH). Additionally, infants born at 25–27 weeks GA with high NfL were at increased risk of developing severe ROP (independent of IVH). NfL was significantly associated with the proteins CDH5, ITGB1, and JAM-A during the first month.

**Conclusion:**

NfL surges after birth in extremely preterm infants, particularly in those with severe IVH and ROP, and in the most immature infants regardless of IVH or ROP severity. These findings suggest NfL as a potential predictor of neonatal morbidities, warranting further validation studies.

**Impact statement:**

- This study shows that higher NfL levels are related to neurovascular morbidities in extremely preterm infants.
- The degree of immaturity seems important as infants born <25 weeks gestational age exhibited high postnatal serum NfL levels irrespective of neurovascular morbidities.
- Our findings suggest a potential link between NfL and neurovascular morbidities possibly affected by a more permeable blood-brain barrier.

## Introduction

Neurofilament light (NfL) forms a part of the neurofilament network located within axons, dendrites, and dendritic spines^1^ and is used as a fluid-based biomarker for brain injury.^1,2^ Research indicates an initial surge in NfL levels post-delivery across very preterm,^3,4^ moderately preterm, and term infants,^4^ correlating with gestational age (GA), birth weight, and Apgar scores.^3,4^ Vaginally delivered infants has shown higher NfL levels post-delivery.^5^ NfL has been recognized as a potential marker for brain and spinal cord injury with a request for specific reference values for the pediatric group from the European Medicines Agency.^6^ Recent reference material has been published for healthy children and adolescent from 0.4 to 22 years of age.^7–9^ However, reference data for preterm infants and term infants for the first month of life are lacking.

NfL levels after preterm birth have been assessed as a potential marker for preterm morbidities, including intraventricular hemorrhage (IVH) and periventricular hemorrhage (IVH1-4), posthemorrhagic ventricular dilatation (PHVD),^4,10,11^ and retinopathy of prematurity (ROP).^3^ Infants with ROP demonstrate an increased risk for compromised neurodevelopment, underscoring the eye-brain connection.^12,13^ Additionally, neonatal NfL levels may correlate with long-term neurodevelopmental outcomes in very and moderate preterm infants.^3,4,11^

The prevalence of IVH is linearly related to the degree of prematurity and commonly occurs during the first days of life. The bleeding appears in the germinal matrix (IVH1) adjacent to the lateral ventricles and may expand into the ventricular system (IVH2-3), and be associated with a periventricular-venous hemorrhagic infarction (PVHI or IVH4) or persisting PHVD.^14,15^ Similarly, ROP, another condition related to prematurity, is diagnosed through retinal examinations typically initiated around 6 weeks after birth.^16^ The severity of ROP is classified by the appearance of a structure at the avascular-vascular junction as stage 1 (demarcation line), stage 2 (ridge), and stage 3 (extraretinal neovascular proliferation or flat neovascularization).^17^

NfL levels in cerebrospinal fluid or blood are thought to reflect neuroaxonal release caused by neuronal damage. Under physiological conditions, NfL is present in cerebrospinal fluid and blood in low concentrations.^2^ Dysfunction in the blood-brain barrier can result in higher NfL levels in the blood.^18,19^ Although blood NfL concentrations are lower than, and only moderately correlated with, cerebrospinal fluid levels, blood has been suggested as a possible alternative biomarker source.^20^ Blood NfL levels are also known to increase with age and age-related comorbidities^21^ and during pregnancy in conditions like preeclampsia.^22^ In children, blood NfL levels decrease with age the first 10 years of life.^8,9^ Blood NfL levels reflect production or release, transport from the central nervous system to the systemic circulation, and normal clearance, which can all be linked to development.

In the context of preterm birth, the loss of omega-3 and omega-6 long-chain polyunsaturated fatty acids (LC-PUFAs) increases the risk for morbidities.^23^ Research on ROP indicates that maintaining sufficient levels of both docosahexaenoic acid (DHA) and arachidonic acid (AA) plays a crucial role in preventing disease.^24^ These fatty acids are essential for brain health, with omega-3 LC-PUFA DHA vital for the survival and differentiation of neuronal retinal photoreceptors.^25^ The Mega Donna Mega Trial demonstrated that combined enteral supplementation with AA and DHA in preterm infants reduced the risk for severe ROP by 50%.^26^ Recently, studies have shown that enteral supplementation with DHA increases intelligence quotient scores in preterm infants^27^ and limits the increase in NfL levels following traumatic brain injury in American football players^28,29^ as well as corresponding injury in a mouse model.^30^

This secondary analysis of the Mega Donna Mega study aims to further explore factors influencing the early postnatal concentrations of NfL and their relationship to ROP and IVH in extremely preterm infants. Thereby the study aims to add to the usefulness of neurological blood biomarkers related to neurovascular morbidities in pediatric clinical practice.

## Methods

### Study design and approval

This study performs a secondary analysis of the Mega Donna Mega randomized controlled trial (ClinicalTrials.gov, NCT03201588),^26^ investigating the effects of enteral lipid supplementation on severe ROP in extremely preterm infants. The study was conducted in accordance with the Declaration of Helsinki^31^ and approved by the University of Gothenburg, Sweden’s Regional Ethical Board (Dnr 303-11, T570-15). Informed consent was obtained from all participating infants’ parents or legal guardians. The Strengthening the Reporting of Observational Studies in Epidemiology (STROBE) reporting guidelines were followed while reporting this secondary analysis.

### Study population

The study included 207 preterm infants born <28 weeks GA, consecutively randomized from December 2016 to August 2019, across three neonatal intensive care units in Sweden. Presence of major malformations was a primary exclusion criterion. Only infants who survived to term equivalent age, with available samples for NfL, IVH, and ROP data were analyzed.

### Exposure

The intervention group received enteral lipid supplementation with 100 mg/kg/day AA and 50 mg/kg/day DHA (Formulaid, DSM), administered from the first three days of life until 40 weeks gestational age. The control group received standard neonatal nutrition.

### Blood sampling and analysis of NfL, fatty acids, and proteins related to the blood-brain barrier

Blood samples (0.6 ml) were collected at birth (from cord blood) and on postnatal days 1, 3, 7, 14, then every second week until postmenstrual week 29, and thereafter at postmenstrual weeks 30, 32, 34, 36, and 40. Samples were processed to coagulate and then centrifuged, with the serum separated and stored at −80 °C.

Serum NfL levels were quantified at the Clinical Neurochemistry, Sahlgrenska University Hospital, using the Simoa NF-Light™ assay on the Simoa HD-X analyzer (Quanterix, Billerica, MA) during spring 2022. Samples were diluted sixfold and run as singlicates. All samples from a specific subject were assembled on the same 96-well microtiter plate, and study subjects were randomly allocated between plates. Two pools of plasma (high [102 pg/mL] and low [14.8 pg/mL] concentration of NfL) were used for quality controls. The intra-assay coefficient of variation (CV) was 4.8% (low level) and 7.5% (high); inter-assay repeatability CV was 4.8% and 8%, respectively. The results for values less than the lowest calibrator (3.41 pg/mL) were set to 1.7 pg/mL.

Phospholipids extracted from serum were derivatized to fatty acid methyl ester and analyzed using gas chromatography-mass spectrometry as earlier described.^32^ To explore proteins related to the blood-brain barrier, 538 proteins analyzed with proximity extension assay coupled to quantitative real-time PCR (OLINK proteomics, Uppsala, Sweden)^33,34^ from six panels INFLAMMATION (v.3021), CARDIOVASCULAR II (v.5006), CARDIOMETABOLIC (v.3603), CARDIOVASCULAR III (v.6113), DEVELOPMENT(v.3512) and METABOLISM (v.3402), were searched by their UniProt GO annotations^35^ for “*tight junc*” AND/OR “*blood-brain*” within biological process, cellular component and molecular function.

Mean daily fatty acids (AA and DHA)^24^ and NfL levels during the first month of life were calculated for each infant. This calculation involved the mean of the area under the curve (AUC) of the respective measurements. For NfL concentration, a minimum of three values, including an early (within the first five postnatal days) and a late measurement (within days 27–33), were required to calculate the AUC. Linear interpolation was allowed for day 28 if two values from adjacent time points were available, provided they were no more than 45 days apart.

### Eye examinations and IVH diagnosis

ROP screenings were conducted following national guidelines. ROP classification follows the International Classification of Retinopathy of Prematurity (ICROP).^16^ IVH diagnosis was based on cranial ultrasound performed at 3, 7 and 21 days postnatal age, with hemorrhages classified according to modified Papile criteria^15,36^ into germinal matrix hemorrhage (IVH1), intraventricular hemorrhage without acute distension of ventricles and with blood filling < 50% (IVH2), intraventricular hemorrhage with acute distension of ventricles and with blood filling > 50% (IVH3) or periventricular venous hemorrhagic infarction (PHVI or IVH4). The presence of PHVD was also recorded.

### Variable definitions and statistical analyses

Variables such as ROP and IVH were defined and categorized for analysis. Standardized birth weight was calculated using the Fenton growth chart,^37^ and infants were grouped by gestational age for statistical considerations.

Samples obtained between postnatal days 12 and 16 were considered as postnatal day 14, while those obtained between postnatal days 21 and 35 were grouped as postnatal day 28.

The Mann‒Whitney U test, Fisher’s exact test, Pearson chi-square test, Mantel‒Haenszel chi-square trend test, and linear and ordinal (checked for proportional odds) and binary logistic regression models were applied. For the description of NfL levels over time for different GA categories, random coefficient models were applied with lognormal distribution, modeling time with the natural cubic splines. Periods with significant differences in NfL levels between the GA categories were adjusted for multiple comparisons using Scheffe’s method.

Data on NfL, AA, and DHA concentrations were log-transformed for normal distribution in statistical models. All tests should be considered exploratory, and two-sided *p*-values < 0.05 indicated statistical significance. Results are presented as relative risks (RR), odds ratios (OR), and their respective 95% confidence intervals (CI). Relationships of the blood-brain barrier and tight junction proteins to NfL levels are described using beta estimates with 95% CI and *p*-value calculated on standardized levels of the proteins, *p*-values were adjusted for false discovery rate (FDR) using the Benjamin–Hochberg method, FDR at 0.05.

Three approaches were used for analyzing NfL levels in regression models: concentrations on postnatal days 14 and 28, respectively, and mean daily levels during the first month. General linear regression models assessed variables significantly associated with NfL levels, such as IVH grade or severity, serum levels of AA and DHA, intervention group, and proteins related to the blood-brain barrier. Logistic regressions were employed for the analysis of ROP outcomes. Models were adjusted for potential impacts of gestational age, study center, and intervention group when appropriate, for ROP models also IVH.

Statistical analyses were performed using SPSS version 29.0 (IBM SPSS Statistics), R-Statistics Software version 4.3.0 (The R Foundation for Statistical Computing), and SAS software version 9.4 (SA Institute., Cary, NC.). The ggplot package in Rstudio or SAS was employed to visualize the data. To identify potential selection bias, infants without data on NfL were compared to those included in the study according to birth characteristics, ROP, and IVH incidence. These comparisons and additional results are presented in the Supplement Appendix (eTable 1).

## Results

### Recruitment

**Fig. 1:**
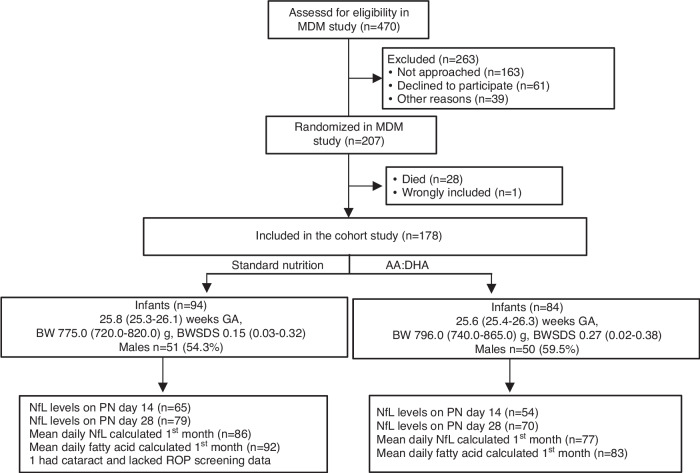
Flowchart of the 178 included infants in this secondary analysis of the Mega Donna Mega study (MDM) cohort. One infant with malformations was identified after initial inclusion and subsequently removed from the analysis. Infants were randomized to enteral supplementation with AA + DHA in 2:1 ratio or standard nutrition. Gestational age (GA), birth weight (BW), and birth weight standard deviation scores (BW-SDS) are presented as medians with 95% confidence intervals. Abbreviations: AA arachidonic acid, BW birth weight, DHA docosahexaenoic acid, GA gestational age, NfL neurofilament light chain, PN postnatal, ROP retinopathy of prematurity, SDS standard deviation score.

One hundred seventy-eight out of 207 (86.0%) infants randomized in the trial were included in this study. The infants had a mean (SD) gestational age of 25.6 (1.4) weeks and birth weight of 806.8 (200) g. Of the 178 infants, 84 were supplemented with AA + DHA, and 94 were on standard nutrition. Birth characteristics were not significantly different between the groups (Fig. 1 and eTable 2).

### The most immature infants have the highest postnatal levels of NfL

**Fig. 2:**
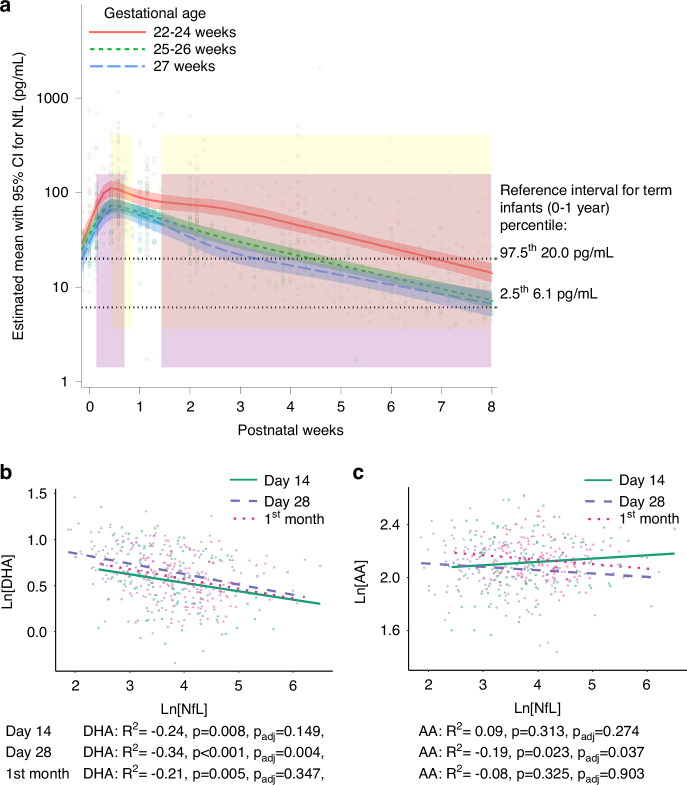
Longitudinal logarithmic levels of serum Neurofilament light (NfL) in relation to gestational age, AA, and DHA. **a** Longitudinal NfL levels illustrated by lines and colors per gestational age group. Lines illustrate the mean concentrations with 95% CIs for each group based on gestational age; shaded areas show the postnatal period with significant differences between groups (yellow, 22–24 vs. 27 weeks GA; pink, 22–24 vs. 25–26 weeks GA). References for healthy infants at one year of age based on Schjørringet al. (2023). The relation to (**b**) docosahexaenoic acid (DHA) and (**c**) arachidonic acid (AA) levels are illustrated in the scatterplots for concentrations at postnatal days 14 (*n* = 119) and 28 (*n* = 149) and for mean daily levels during the first month (*n* = 162). *P*-values were adjusted for gestational age and intervention group. Abbreviations: AA arachidonic acid, CI Confidence interval, DHA docosahexaenoic acid, GA gestational age, NfL neurofilament light chain.

NfL concentrations were analyzed in relation to the degree of immaturity and infants were grouped by GA at birth, intervention and morbidities (Table 1). Fifty-seven infants were born <25 weeks GA, 81 at 25–26 weeks GA, and 40 at 27 weeks GA. All three groups showed an increase in NfL after birth, followed by declining levels after ~1 week of postnatal age. The infants born before 25 weeks GA showed significantly higher NfL levels during days 1–5 and 10–56 compared to those born at week 27 (Fig. 2a).

**Table 1:** NfL concentrations at days 14 and 28 and mean daily levels in the first month of life.

Medians (95% CI), [range] pg/mL, *n*.			
Infant group	NfL day 14	NfL day 28	Mean NfL/day 1st month ^a^
All (*n* = 178)	45.1 (38.6–51.2) [11.3–663.0], *n* = 119	24.1 (20.1–29.9) [6.5–1057.9], *n* = 149	55.0 (44.1–60.9) [11.9–435.2], *n* = 163
22–25 weeks GA (*n* = 57)	72.7 (52.5–86.4) [19.6–663.0], *n* = 38	41.5 (32.3–54.6) [12.7–501.0], *n* = 48	77.8 (66.6–94.2) [22.3–285.9], *n* = 54
25–26 weeks GA (*n* = 81)	37.4 (31.0–48.7) [11.3–365.0], *n* = 57	18.5 (15.9–23.2) [7.5–402.0], *n* = 69	43.6 (40.2–55.6) [12.9–144.9], *n* = 73
27 weeks GA (*n* = 40)	24.4 (19.8–42.8) [14.6–282.0], *n* = 24	12.7 (10.5–19.9) [6.5–1057.9], *n* = 32	37.6 (28.4–43.7) [11.9–435.2], *n* = 36
Standard nutrition (*n* = 94)	48.1 (40.7–55.1) [11.3–663.0], *n* = 65	25.7 (21.5–35.2) [6.5–1057.9], *n* = 79	64.1 (55.8–73.9) [12.9–435.2], *n* = 86
AA + DHA supplemented (*n* = 84)	41.3 (32.0–50.2) [17.1–365.0], *n* = 54	21.4 (16.0–29.2) [6.9–501], *n* = 70	44.2 (42.6–55.0) [11.9–261.7], *n* = 77
IVH4 (*n* = 12)	115.0 (81.2–246.0) [40.7–663.0], *n* = 8	46.8 (24.1–70.7) [13.4–73.3], *n* = 12	105.5 (69.3–117.5) [60.2–285.9], *n* = 11
IVH3 (*n* = 9)	51.0 (35.4–71.6) [31.2–200.0], *n* = 8	36.2 (20.3–98.7) [14.6–113.0], *n* = 8	74.4 (58.0–91.8) [40.3–140.6], *n* = 9
IVH2 (*n* = 25)	54.6 (50.3–181.0) [17.1–365.0], *n* = 21	38.0 (26.9–56.7) [6.9–218.0], *n* = 23	94.2 (66.6–124.4) [22.3–261.7], *n* = 23
IVH1 (*n* = 24)	28.8 (24.5–43.4) [17.9–126.0], *n* = 16	19.9 (12.2–35.2) [7.4–501.0], *n* = 20	43.3 (39.1–86.0) [20.5–241.3], *n* = 21
No IVH (*n* = 108)	39.7 (30.8–46.1) [11.3–144.0], *n* = 66	19.0 (15.9–23.7) [6.5–1057.9], *n* = 86	43.7 (39.0–54.2) [11.9–435.2], *n* = 99
Severe ROP (stage 3 and type 1) (*n* = 50)	55.1 (48.1–73.2) [19.1–663.0], *n* = 35	36.1 (26.9–51.9) [9.9–501.0], *n* = 43	72.0 (59.9–93.6) [22.3–285.9], *n* = 47
Mild/moderate ROP (stage 1 and 2) (*n* = 55)	34.0 (28.5–50.2) [16.1–183.0], *n* = 38	20.0 (17.3–30.4) [7.5–1057.9], *n* = 45	55.0 (44.1–66.5) [16.5–435.2], *n* = 51
No ROP (*n* = 72)	42.8 (26.3–50.3) [11.3–365.0], *n* = 45	19.3 (14.2–25.7) [6.5–108.0], *n* = 60	41.8 (36.8–49.1) [11.9–261.9], *n* = 64

*AA* Arachidonic acid, *CI* confidence interval, *DHA* Docosahexaenoic acid, *GA* gestational age, *IVH* intraventricular hemorrhage, *NfL* neurofilament light chain, *ROP* retinopathy of prematurity.

^a^Calculated as the mean of the area under the curve (AUC) of the concentrations during the first month of life.

### Systemic levels of AA and DHA correlated negatively with NfL levels

On postnatal day 28, AA and DHA were significantly negatively associated with NfL. However, not significant after adjustment on day 14 or for mean daily levels (Fig. 2b, c). NfL levels did not differ between infants receiving the LC-PUFA supplement and the controls during the first month (eTable 3).

### NfL levels increase with IVH grade

Investigation revealed a significant relationship for higher NfL and increased IVH severity (mean NfL/day first month: *p*_adj_ = 0.002), notably higher for IVH 2 and 4 against no-IVH (eTable 4). Comparing the binary groups (no IVH/IVH1 vs. IVH2-4) also showed an increased RR for higher NfL and ROP severity (mean NfL/day first month: _adj_RR [95% CI] 1.50 [1.22–1.84], *p*_adj_ < 0.001). Similar results were found for NfL concentrations at postnatal day 14 (eTable 4).

### NfL levels and ROP severity

Ordinal regression showed an association between NfL and ROP; however, adjustment negated significance (mean NfL/day first month: no-, mild/moderate [stage 1–2], and severe ROP [stage 3 or treated]) (OR [95% CI] 2.57 [1.59–4.14], *p* < 0.001). Results were similar with severe ROP as outcome in binary regression (eTable 5).

### The most immature infants have high NfL levels irrespective of IVH grade and ROP severity

**Fig. 3:**
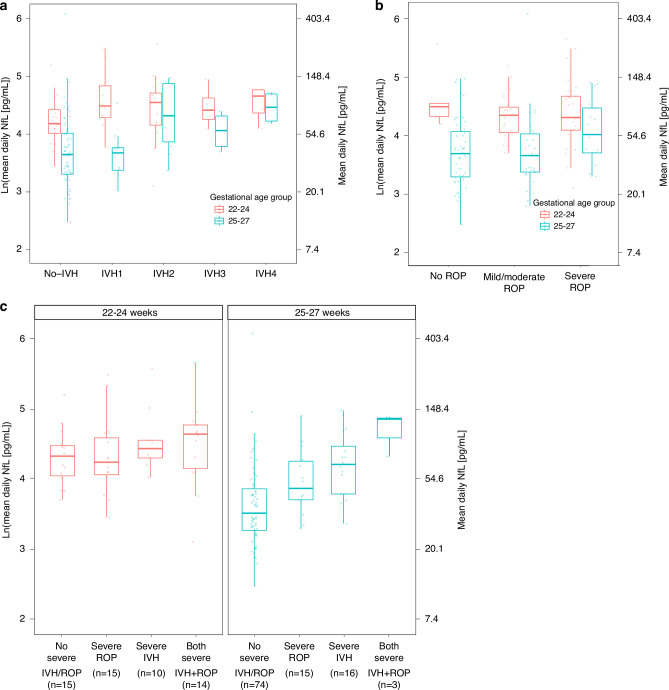
Mean daily NfL levels associate with neurovascular morbidities, but only in infants born ≥ 25 weeks GA. Serum NfL levels are presented as the natural logarithm on the y-axis and with the translation to pg/mL on the right y-axis according to gestational age groups (colors), for (**a**) infants based on IVH grade, and (**b**) infants based on ROP severity (**c**) infants without severe ROP (no/mild/moderate ROP) and IVH (no IVH or IVH1), infants with severe ROP but no severe IVH (IVH2-4), infants with severe IVH and no severe ROP and infants with both severe ROP and IVH. Abbreviations: NfL neurofilament light chain, IVH intraventricular hemorrhage: ROP retinopathy of prematurity.

Subgroup analysis underscores consistently high NfL levels in the most preterm infants (<25 weeks GA), regardless of IVH grade or ROP severity (Fig. 3a–c). In contrast, those born at 25–27 weeks GA with severe IVH displayed a notable risk for higher NfL (eTable 6). In addition, infants with higher mean NfL/day first month showed an increased risk for severe ROP, independent of severe hemorrhage (IVH2-4, _adj_OR [95% CI]: 2.62 [1.06–6.51], *p*_adj_ = 0.038), with similar results at day 28 (eTable 7).

### Blood-brain barrier and NfL levels

**Fig. 4:**
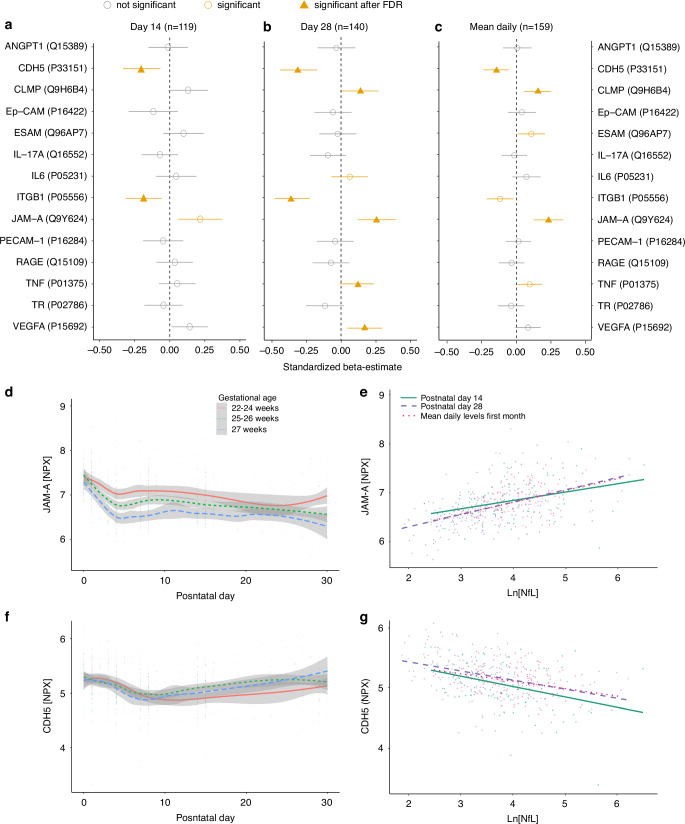
Association between levels of serum proteins related to the blood-brain barrier and Neurofilament light. General linear regression with naturally log-transformed NfL levels as the dependent variable to explore the relationship to the proteins related to the blood-brain barrier and/or tight junctions (as standardized values to enable comparison), illustrated for (**a**) postnatal day 14 (*n* = 119), (**b**) postnatal 28 (*n* = 140) and (**c**) mean daily first month levels (*n* = 156). For JAM-A (**d**) and CDH5 (**f**), the longitudinal development is illustrated for groups based on gestational ages. Lines illustrate the mean concentrations with 95% CIs by smoothed conditional means using the LOESS function in ggplot. Correlations between NfL levels and JAM-A (**e**) and CDH5 (**g**) are illustrated for each time variable. Abbreviations: AA Arachidonic acid, ANGPT1 angiopoietin-1, CDH5 cadherin-5, CLMP CXADR-like membrane protein, DHA Docosahexaenoic acid, Ep-CAM epithelial cell adhesion molecule, ESAM endothelial cell-selective adhesion molecule, IL-6 interleukin-6, IL-17A interleukin-17A, ITGB1 integrin beta-1, JAM-A junctional adhesion molecule A, NPX normalized protein expression (arbitrary unit on a log-2 scale), PECAM platelet endothelial cell adhesion molecule, RAGE advanced glycosylation end product-specific receptor, TNF tumor necrosis factor, VEGF-A vascular endothelial growth factor, TR transferrin receptor protein

Out of the 538 proteins we specifically analyzed 14 proteins annotated related to tight junctions and/or blood-brain barrier in relation to NfL (Fig. 4a–c). Junctional adhesion molecule A (JAM-A) was positively associated, and Cadherin-5 (CDH5) and Integrin beta-1 (ITGB1) negatively, with NfL levels at postnatal days 14 and 28 and as mean daily levels (Fig. 4d–f).

## Discussion

This secondary analysis confirmed a relationship between NfL levels and IVH in extremely preterm infants, noting a significant NfL increase post-birth, particularly in the most immature infants. NfL levels surpassed the 97.5th percentile of healthy one-year-olds^7^ within the first month. Intriguingly, the most immature infants (<25 weeks GA) showed high NfL levels regardless of grades of IVH or ROP, however for infants at higher GA (25–27 weeks) a relationship with ROP was observed.

Blood NfL levels have been associated with a disrupted blood-brain barrier in adults,^18,19^ and tight junction proteins have been reported as promising markers for cerebral vascular dysfunction in neonatal brain injuries.^38^ Our study revealed a positive association between JAM-A and NfL levels, with higher postnatal levels in the most immature infants. JAM-A is a transmembrane glycoprotein^39^ that plays a role in the tight junction formation.^39,40^ Higher levels of JAM-A in more preterm infants might be explained by a reduced integrity in the blood-brain barrier as described for other proteins involved in the tight junction.^38^ Interestingly, CDH5, also known as VE-cadherin, and ITGB1, was negatively associated with NfL and exhibited rising postnatal levels from day 10, with a slower increase in the most immature infants. CDH5 is a major component of adherens junctions and vital for vessel integrity. Lower levels of CDH5 may indicate affected vessel permeability^41,42^ and ITGB1 is closely related to CDH5.^43^

Our previous finding in another extremely preterm cohort indicated high early NfL levels in infants who later developed ROP.^3^ This study adds more evidence suggesting an association between severe ROP and serum NfL in the first postnatal month for infants born ≥25 weeks GA. NfL levels have been described to reflect inner retinal atrophy and neuroaxonal damage, in conditions like neurodegeneration,^44^, and multiple sclerosis.^45^ In addition, higher NfL levels were observed in children with acquired demyelination syndromes compared to infants with and without other neurological diseases.^46,47^ However, NfL levels in blood during the first years of life are affected by production, release, transportation and clearance and can thus reflect several conditions.

Concerning IVH, our previous study^3^ did not strongly link NfL with IVH, likely due to limited data on specific IVH grades. With more detailed data in this cohort, we observed a link between higher NfL levels in the initial postnatal month and more severe IVH (2-4) in infants ≥25 weeks GA. Goeral et al. ^11^ also showed high levels of NfL at the diagnosis of peri/intraventricular hemorrhage in preterm infants, which decreased markedly at term equivalent age. Interestingly, infants with IVH grade 1 did not exhibit elevated NfL levels relative to those without IVH. In a preterm rabbit model, IVH with hemorrhage restricted to the ventricular system was accompanied by increased pro-inflammatory cytokines in periventricular white matter tracts caused by extracellular hemoglobin.^48,49^ These translational findings support that elevated NfL levels reflect neuronal injury associated with IVH grades 2–4.

IVH is a significant predictor of adverse neurodevelopmental outcomes in preterm infants.^50^ While grades 1 and 2 IVH indicate the presence of blood within the germinal matrix or ventricles, they are generally associated with a better prognosis compared to grades 3 and 4 IVH. Grade 3 IVH involves more extensive bleeding with ventricular dilation, and grade 4 IVH includes parenchymal hemorrhage, both of which lead to more severe neurodevelopmental impairments due to the associated brain tissue damage and inflammation. This classification is supported by existing literature and clinical observations.^51^ Hence, our grouping of IVH grades has been done in accordance with these findings to reflect the severity and potential outcomes accurately.

Of note, the apparent relationship between the degree of immaturity at birth and circulating NfL obscured the relationship between severe IVH and NfL in infants born at <25 weeks GA. Whether higher serum NfL, irrespective of IVH and ROP, represents white matter abnormality or constitutes an expression of immaturity without neurodevelopmental relevance in the most immature infants remains to be shown.

The relationship between LCPUFAs and NfL has been sparsely discussed.^28,52,53^ Given an association between ROP and IVH^54^ and the increased risk of neurodevelopmental impairment,^55–57^ coupled with NfL’s status as a marker for neuronal injury,^58^ it’s critical to question how AA + DHA supplementation might influence neurodevelopment. Our previous research revealed a higher number of infants with visual acuity ≥ 20/63 at 2.5 years after AA + DHA supplementation.^59^ Ongoing plans include monitoring this cohort’s visual (morphological and functional), neurodevelopmental, and neuropsychiatric outcomes as they approach early school age. Furthermore, regarding AA + DHA supplementation and NfL levels, we intend to explore the connection to structural changes in the retina through Optical Coherence Tomography (OCT), as Ingvaldsen et al. ^60^ suggested. We also plan to further investigate proteins linked to NfL, associated to brain development and visual outcome in this cohort.

### Strengths and limitations

Our study meticulously explored the relationship between early NfL concentrations, IVH, and ROP. Adopting three distinct approaches for NfL levels aiming to analyze NfL’s longitudinal evolution and concentrations at specific time points, thus bolstering our findings. In addition, the high-temporal-resolution assessments of NfL, AA and DHA in this unique population using state-of-the-art analytical techniques and detailed clinical data in line with the Mega Donna Mega study protocol strengthens the interpretation.

The number of available NfL concentrations to analyze against IVH and ROP was incomplete due to limited blood sampling. For ethical reasons, blood was taken in connection to clinical sampling. Infants without samples tended to be born at slightly higher gestational ages, had fever days with respiratory support, and showed a lower frequency of IVH. We cannot rule out that the results for IVH are affected by missing data (eTable 1). A risk of selection bias for the population is that, for various reasons, not all eligible infants meeting the inclusion criteria were included in the study.

Previous findings on NfL, IVH, and ROP focused on infants <32 weeks GA.^3^ Early postnatal NfL levels strongly correlate with GA and the most immature infants have both higher NfL levels and a greater risk of ROP and IVH. Therefore, the absence of very preterm infants (28–32 weeks GA) in the current cohort might explain the weaker association between NfL and ROP.

## Conclusion

This study confirms that NfL surges in blood following birth in extremely preterm infants with higher levels in infants with severe IVH and severe ROP among infants at higher GA. Interestingly, the most immature infants (<25 weeks GA) exhibited high NfL independently of IVH or ROP severity. Our findings may help to predict neonatal morbidities in a vulnerable group of extremely preterm infants, but further studies are needed to validate NfL as a clinical biomarker. In addition, NfL levels seem to correlate with biomolecules associated with the blood-brain barrier

## Supplementary information

Strobe statement

Supplement appendix

## Data Availability

The datasets generated and/or analyzed during the current study are not publicly available due to ethical permits and The General Data Protection Regulation (GDPR) Regulation (EU) 2016/679 on the protection of natural persons with regard to the processing of personal data and on the free movement of such data law regulates the availability of personal data, but deidentified data are available from the corresponding author on reasonable request.
