# Supplementary material for: Neurofilament light chain associates with IVH and ROP in extremely preterm infants
Source: Pediatr Res. 2024 Sep 24;97(3):1183–92. doi: 10.1038/s41390-024-03587-5 (PMC12055587; doi:10.1038/s41390-024-03587-5)
Supplement: Supplementary file 1 — Strobe statement [file 41390_2024_3587_MOESM1_ESM.pdf]

STROBE Statement—checklist of items that should be included in reports of observational studies

	Item No.	Recommendation	Page No.	Relevant text from manuscript
**Title and abstract**	1	(*a*) Indicate the study's design with a commonly used term in the title or the abstract	3	Line 56
		(*b*) Provide in the abstract an informative and balanced summary of what was done and what was found	3	
**Introduction**				
Background/rationale	2	Explain the scientific background and rationale for the investigation being reported	4-5	
Objectives	3	State specific objectives, including any prespecified hypotheses	5	Lines 116-118
**Methods**				
Study design	4	Present key elements of study design early in the paper	6	Lines 121-124
Setting	5	Describe the setting, locations, and relevant dates, including periods of recruitment, exposure, follow-up, and data collection	6	Lines 130-131, 135-137, 138-141
Participants	6	(*a*) *Cohort study*—Give the eligibility criteria, and the sources and methods of selection of participants. Describe methods of follow-up<br>*Case-control study*—Give the eligibility criteria, and the sources and methods of case ascertainment and control selection. Give the rationale for the choice of cases and controls<br>*Cross-sectional study*—Give the eligibility criteria, and the sources and methods of selection of participants	6	Lines 130-133
		(*b*) *Cohort study*—For matched studies, give matching criteria and number of exposed and unexposed<br>*Case-control study*—For matched studies, give matching criteria and the number of controls per case	NA	
Variables	7	Clearly define all outcomes, exposures, predictors, potential confounders, and effect modifiers. Give diagnostic criteria, if applicable	6-8	Lines 134-137, 163-171, 173-177, 195-197
Data sources/ measurement	8*	For each variable of interest, give sources of data and details of methods of assessment (measurement). Describe comparability of assessment methods if there is more than one group	*6-7*	Lines: 138-162
Bias	9	Describe any efforts to address potential sources of bias	8 and eTable 1	Lines 201-203,
Study size	10	Explain how the study size was arrived at	6	Lines 132-133

Continued on next page

Quantitative variables	11	Explain how quantitative variables were handled in the analyses. If applicable, describe which groupings were chosen and why	7-8	Lines 184-186
Statistical methods	12	(*a*) Describe all statistical methods, including those used to control for confounding	7-8	Lines 178-183, 187-190
		(*b*) Describe any methods used to examine subgroups and interactions	7	Lines 173-175
		(*c*) Explain how missing data were addressed	7	Lines 157-162, 176-177
		(*d*) *Cohort study*—If applicable, explain how loss to follow-up was addressed<br>*Case-control study*—If applicable, explain how matching of cases and controls was addressed<br>*Cross-sectional study*—If applicable, describe analytical methods taking account of sampling strategy	NA	
		(*e*) Describe any sensitivity analyses	NA	
**Results**				
Participants	13*	(a) Report numbers of individuals at each stage of study—eg numbers potentially eligible, examined for eligibility, confirmed eligible, included in the study, completing follow-up, and analysed	9 and Figure 1 and Table 1	Line 206
		(b) Give reasons for non-participation at each stage	Figure 1	
		(c) Consider use of a flow diagram	Figure 1	
Descriptive data	14*	(a) Give characteristics of study participants (eg demographic, clinical, social) and information on exposures and potential confounders	9, Figure 1 and eTable 2	Lines 205-209
		(b) Indicate number of participants with missing data for each variable of interest	eTable 1	
		(c) *Cohort study*—Summarise follow-up time (eg, average and total amount)	Table 1 and Figure 1	
Outcome data	15*	*Cohort study*—Report numbers of outcome events or summary measures over time	Table 1	
		Case-control study—Report numbers in each exposure category, or summary measures of exposure		
		Cross-sectional study—Report numbers of outcome events or summary measures		
Main results	16	(*a*) Give unadjusted estimates and, if applicable, confounder-adjusted estimates and their precision (eg, 95% confidence interval). Make clear which confounders were adjusted for and why they were included	9	Lines 221-231, Figure 4<br>Is fully reported in supplement appendix eTable 4-7
		(*b*) Report category boundaries when continuous variables were categorized	NA	
		(*c*) If relevant, consider translating estimates of relative risk into absolute risk for a meaningful time period	NA	

Continued on next page

Other analyses	17	Report other analyses done—eg analyses of subgroups and interactions, and sensitivity analyses	9-10	Lines 232-238
**Discussion**				
Key results	18	Summarise key results with reference to study objectives	11	Lines 246-250
Limitations	19	Discuss limitations of the study, taking into account sources of potential bias or imprecision. Discuss both direction and magnitude of any potential bias	12	Lines 299-306
Interpretation	20	Give a cautious overall interpretation of results considering objectives, limitations, multiplicity of analyses, results from similar studies, and other relevant evidence	11-12	Lines 251-261. 262-266, 267-276, 282-287
Generalisability	21	Discuss the generalisability (external validity) of the study results	11-12	Lines 276-281, 287-291
**Other information**				
Funding	22	Give the source of funding and the role of the funders for the present study and, if applicable, for the original study on which the present article is based	18	

*Give information separately for cases and controls in case-control studies and, if applicable, for exposed and unexposed groups in cohort and cross-sectional studies.

Note: An Explanation and Elaboration article discusses each checklist item and gives methodological background and published examples of transparent reporting. The STROBE checklist is best used in conjunction with this article (freely available on the Web sites of PLoS Medicine at http://www.plosmedicine.org/, Annals of Internal Medicine at http://www.annals.org/, and Epidemiology at http://www.epidem.com/). Information on the STROBE Initiative is available at www.strobe-statement.org.
