# Supplementary material for: Neurofilament light chain associates with IVH and ROP in extremely preterm infants
Source: Pediatr Res. 2024 Sep 24;97(3):1183–92. doi: 10.1038/s41390-024-03587-5 (PMC12055587; doi:10.1038/s41390-024-03587-5)
Supplement: Supplementary file 2 — Supplement appendix [file 41390_2024_3587_MOESM2_ESM.pdf]

Supplemental Appendix to

Neurofilament Light Chain associates with IVH and ROP in Extremely Preterm Infants: Insights from the Mega Donna Mega Study

Ulrika Sjöbom[1,2*], Annika Öhrfelt[3], Aldina Pivodic[2], Anders K Nilsson[2], Kaj Blennow[3,4,5, 6], Henrik Zetterberg[3,4,7,8,9,10], William Hellström[11], Hanna Danielsson[12,13], Lotta Gränse[14], Karin Sävman[11,15], Dirk Wackernagel[16,17], Ingrid Hansen-Pupp[18], David Ley[18], Ann Hellström[2], Chatarina Löfqvist[1,2]

[1]Learning and Leadership for Health Care Professionals, Institute of Health and Care Science at Sahlgrenska Academy, University of Gothenburg, Gothenburg, Sweden

[2]Department of Clinical Neuroscience, Institute of Neuroscience and Physiology, Sahlgrenska Academy, University of Gothenburg, Gothenburg, Sweden

[3]Department of Psychiatry and Neurochemistry, Institute of Neuroscience and Physiology, The Sahlgrenska Academy at the University of Gothenburg, Mölndal, Sweden

[4]Clinical Neurochemistry Laboratory, Sahlgrenska University Hospital, Mölndal, Sweden

[5]Paris Brain Institute, ICM, Pitié-Salpêtrière Hospital, Sorbonne University, Paris, France

[6]Neurodegenerative Disorder Research Center, Division of Life Sciences and Medicine, and Department of Neurology, Institute on Aging and Brain Disorders, University of Science and Technology of China and First Affiliated Hospital of USTC, Hefei, P.R. China

[7]Department of Neurodegenerative Disease, University College of London Institute of Neurology, London, United Kingdom

[8]UK Dementia Research Institute, University College of London, London, United Kingdom

[9]Hong Kong Center for Neurodegenerative Diseases, Clear Water Bay, Hong Kong, China

[10]Wisconsin Alzheimer's Disease Research Center, University of Wisconsin School of Medicine and Public Health, University of Wisconsin-Madison, Madison, WI, USA

[11]Department of Pediatrics, Institute of Clinical Sciences, Sahlgrenska Academy, University of Gothenburg, Gothenburg, Sweden

[12] Centre for Translational Microbiome Research, Department of Microbiology, Tumor and Cell Biology, Karolinska Institutet, Stockholm, Sweden

[13] Sach's Children's and Youth Hospital, Södersjukhuset, Stockholm, Sweden.

[14]Department of Clinical Sciences, Ophthalmology, Skåne University Hospital, Lund University, Lund, Sweden

[15]Region Västra Götaland, Department of Neonatology, The Queen Silvia Children's Hospital, Sahlgrenska University Hospital, Gothenburg, Sweden

[16]Department of Clinical Science, Intervention and Technology (CLINTEC), Karolinska Institutet, Stockholm, Sweden

[17]Division of Neonatology, Department of Pediatrics, University Medical Center of the Johannes Gutenberg-University Mainz, Mainz, Germany

[18]Department of Clinical Sciences Lund, Pediatrics, Lund University, Skåne University Hospital, Lund, Sweden

***Corresponding author**: Ulrika Sjöbom, Box 457, 40530 Göteborg, phone: +46-709995166, ulrika.sjobom@gu.se

Content

eTable 1 Comparison of infants without NfL concentration data with infants included in the study 3
eTable 2 Description of the 178 infants from the Mega Donna Mega cohort compared by intervention group 5
eTable 3. NfL concentrations (pg/mL) between the AA+DHA-supplemented and standard nutrition groups were compared 6
eTable 4 Relative risk (95% confidence interval) per IVH grade 7
eTable 5. Ln NfL concentrations (pg/mL) according to ROP severity and severe ROP 8
eTable 6. Ln NfL concentrations (pg/mL) according to severe IVH (IVH2-4) and gestational age group (<25/≥25 weeks) 9
eTable 7. Ln NfL concentrations (pg/mL) according to severe ROP and gestational age group (<25/≥25 weeks GA) 10

eTable 1 Comparison of infants without NfL concentration data with infants included in the study

	Missing mean daily NfL the first month**	Available mean daily NfL the first month**	P*	Missing NfL at day 14	Available NfL at day 14	P*	Missing NfL at day 28	Available NfL at day 28	P*
**Number of infants**	15	163		59	119		29	149	
**AA+DHA, n (%)**	7 (46.7)	77 (47.2)	1.000	30 (50.8)	54 (45.4)	0.526	14 (48.3)	70 (47.0)	1.000
**Standard nutrition, n(%)**	8 (53.3)	86 (52.8)		29 (49.2)	65 (54.6)		15 (51.7)	79 (53.0)	
**GA <25 weeks**	3 (20.0)	54 (33.1)	0.371	19 (32.2)	38 (31.9)	0.522	9 (31.0)	48 (32.2)	0.762
**GA 25-26 weeks**	8 (53.3)	73 (44.8)		24 (40.7)	57 (47.9)		12 (41.4)	69 (46.3)	
**GA 27 weeks**	4 (26.7)	36 (22.1)		16 (27.1)	24 (20.2)		8 (27.6)	32 (21.5)	
**No ROP, n(%)**	8 (53.3)	64 (39.5)	0.304	27 (45.8)	45 (38.1)	0.366	12 (41.4)	60 (40.5)	0.731
**Mild/moderate ROP, n(%)**	4 (26.7)	51 (31.5)		17 (28.8)	38 (32.2)		10 (34.5)	45 (30.4)	
**Severe ROP, n(%)**	3 (20.0)	47 (29.0)		15 (25.4)	35 (29.7)		7 (24.1)	43 (29.1)	
**No IVH, n(%)**	9 (60.0)	99 (60.7)	0.735	42 (71.2)	66 (55.5)	0.065	22 (75.9)	86 (57.7)	**0.030**
**IVH1, n(%)**	3 (20.0)	21 (12.9)		8 (13.6)	16 (13.4)		4 (13.8)	20 (13.4)	
**IVH2, n(%)**	2 (13.3)	23 (14.1)		4 (6.8)	21 (17.6)		2 (6.9)	23 (15.4)	
**IVH3, n(%)**	0 (0.0)	9 (5.5)		1 (1.7)	8 (6.7)		1 (3.4)	8 (5.4)	
**IVH4, n(%)**	1 (6.7)	11 (6.7)		4 (6.8)	8 (6.7)		0 (0.0)	12 (8.1)	
**Median gestational age at birth (95% CI of the median) [range]**	26.1, (25.6-27.1) [23.0-27.7]	25.6, (25.3-26.1) [22.6-27.9]	0.325	26.1, (25.6-26.7) [22.9-27.8]	25.6, (25.3-26.1) [22.6-27.9]	0.108	26.1, (25.3-26.9) [22.9-27.9]	25.6, (25.4-26.1) [22.6-27.9]	0.308
**Median birth weight(95% CI of the median) [range]**	866.0 (640.0-995.0) [534.0-1120.0]	785.0 (745.0-825.0) [425.0-1345.0]	0.836	805.0 (730.0-870.0) [470.0-1345.0]	770.0 (735.0-825.0) [425.0-1330.0]	0.536	790.0 (698.0-965.0) [470.0-1120.0]	785.0 (745.0-827.0) [425.0-1345.0]	0.873
**Median birth weight SDS (Fenton) (95% CI of the median) [range]**	0.10, (-0.2-0.7) [-1.9-0.8]	0.20, (0.1-0.3) [-2.3-2.3]	0.531	0.13 (0.06-0.36) [-1.19-2.34]	0.18, (0.03-0.36) [-2.25-1.81]	0.647	0.13, (-0.1-0.6) [-1.97-1.61]	0.18, (0.1-0.3) [-2.25-2.34]	0.886
**Median Apgar score at 5 minutes (95% CI of the median) [range]**	7.0, (6.0-10.0) [5.0-10.0]	7.0, (7.0-8.0) [1.0-10.0]	0.768	8.0, (8.0-9.0) [2.0-10.0]	7.0, (7.0-8.0) [1.0-10.0]	0.080	8.0, (8.0-9.0) [4.0-10.0]	7.0, (7.0-8.0) [10-10.0]	0.274
**Median number of days with respiratory support (95% CI of the median) [range]**	67.0, (53.0-101.0) [4.0-299.0]	73.0, (68.0-79.0) [5.0-281.0]	0.973	67.0, (56.0-78.0) [5.0-299.0]	74.0, (68.0-80.0) [4.0-191.0]	0.278	63.5, (55.0-78.0) [4.0-299.0]	73.0, (68.0-79.0) [5.0-281.0]	0.353
**Median number of days with 02 (95% CI of the median) [range]**	95.0, (77.0-109.0) [2.0-299.0]	85.0, (78.0-96.0) [0.0-291.0]	0.959	77.0, (67.0-100.0) [0.0-299.0]	86.0, (80.0-97.0) [0.0-291.0]	0.343	75.0, (65.0-98.0) [4.0-299.0]	85.0, (80.0-97.0) [0.0-291.0]	0.311
**Males n (%)**	9 (60.0)	92 (56.4)	1.000	30 (50.8)	71 (59.7)	0.335	16 (55.2)	85 (57.0)	1.000
**Twins/triplets n (%)**	2 (13.3)	31 (19.1)	0.780	9 (15.5)	24 (20.2)	0.540	6 (20.7)	27 (18.2)	0.795
**Center 1 n (%)**	2 (13.3)	63 (38.7)	0.113	14 (23.7)	51 (42.9)	**0.033**	7 (24.1)	58 (38.9)	0.125
**Center 2 n (%)**	5 (33.3)	48 (29.4)		23 (39.0)	30 (25.2)		13 (44.8)	40 (26.8)	
**Center 3 n (%)**	8 (53.3)	52 (31.9)		22 (37.3)	38 (31.9)		9 (31.0)	51 (34.2)	
**Vaginal delivery**	7 (46.7)	63 (38.7)	0.587	21 (35.6)	49 (41.2)	0.517	12 (41.4)	58 (38.9)	0.837

Abbreviations: AA: arachidonic acid, DHA: docosahexaenoic acid, AA+DHA: enteral supplementation with AA and DHA in a ratio of 2:1, CI: confidence interval, GA: gestational age, NfL: neurofilament light chain, ROP: retinopathy of prematurity, SD: standard deviation, SDS: standard deviation score.

** P values were calculated against the group with NfL values available at the defined timepoint using the Mann–Whitney U test for continuous data, Fisher's exact test to compare two groups, the Mantel–Haenszel chi-square trend test to compare odered categorical variables (ROP severity groups) and the Pearson chi-square test for nonordered categorical variables (center). P-values <0.05 are bold.*

**Calculated as the mean of the area under the curve (AUC) of the concentrations during the first month of life

eTable 2 Description of the 178 infants from the Mega Donna Mega cohort compared by intervention group

	AA+DHA supplementation n=84	Standard nutrition N=94	Total N=178	P value*
**GA at birth in weeks, mean (SD), median (95% CI of the median, range)**	25.6 (1.5)<br>25.6 (25.4-26.3, 22.6-27.9)	25.6 (1.4)<br>25.8 (25.3-26.1, 22.9-27.9)	25.6 (1.4)<br>25.6 (25.4-26.1, 22.6-27.9)	0.705
**GA <25 weeks**	26 (30.9)	31 (39.4)	57 (32.0)	0.536
**GA 25-26 weeks**	37 (44.0)	44 (46.8)	81 (45.5)	
**GA 27 weeks**	21 (38.9)	19 (20.2)	40 (22.5)	
**Birth weight (g), mean (SD), median (95% CI of the median, range)**	818 (205)<br>796 (740-865, 455-1345)	795 (195)<br>775 (720-820, 425-1330)	806 (200)<br>788 (745-825, 425-1345)	0.434
**Birth weight SDS (Fenton), mean (SD), median (95% CI of the median, range)**	0.15 (0.84)<br>0.27 (0.02-0.38, -2.25-2.34)	0.06 (0.8)<br>0.15 (0.03-0.32, -2.05-1.81)	0.1 (0.8)<br>0.18 (0.07-0.32, -2.25-2.34)	0.425
**Apgar score at 5 minutes, mean (SD), median (95% CI of the median, range)**	7.4 (1.9)<br>8 (8-9, 2-10)	7.1 (2.1)<br>7 (7-8, 1-10)	7.2 (2.0)<br>7 (7-8, 1-10)	0.423
**Number of days with respiratory support, mean (SD), median (95% CI of the median, range), (N=173),**	79.3 (49.3)<br>72 (64-80, 5-299)	71.1 (28.7)<br>71.5 (63-78, 4-190)	75.0 (39.9)<br>72 (67-78, 4-299)	0.783
**Number of days with 02 mean (SD), median (95% CI of the median, range), (N=173)**	102.4 (61.6)<br>89.5 (78-106, 0-299)	93.6 (59.4)<br>81.5 (72-95, 2-291)	97.8 (60.4)<br>85 (80-96, 0-299)	0.163
**Severe ROP, (stage 3 and type 1) n (%)**	16 (19.0)	34 (36.6)	50 (28.2)	0.118
**Mild/Moderate ROP, (stage 1 and 2) n (%)**	33 (39.3)	22 (23.7)	55 (31.1)	
**No ROP n (%)**	35 (41.7)	37 (39.8)	72 (40.7)	
**IVH4, n(%)**	3 (3.6)	9 (9.6)	12 (6.7)	
**IVH3, n(%)**	4 (4.8)	5 (5.3)	9 (5.1)	
**IVH2, n(%)**	13 (15.5)	12 (12.8)	25 (14.0)	0.445
**IVH1, n(%)**	14 (16.7)	10 (10.6)	24 (13.5)	
**No IVH n(%)**	50 (59.5)	58 (61.7)	108 (60.7)	
**Males n (%)**	50 (59.5)	51 (54.3)	101 (56.7)	0.545
**Twins/triplets n (%)**	14 (16.9)	19 (20.2)	33 (18.6)	0.699
**Center 1 n (%)**	32 (38.1)	33 (35.1)	65 (36.5)	0.894
**Center 2 n (%)**	25 (29.8)	28 (29.8)	53 (29.8)	
**Center 3 n (%)**	27 (32.1)	33 (35.1)	60 (33.7)	
**Vaginal delivery n (%)**	32 (38.1)	38 (40.4)	70 (39.3)	0.761

Abbreviations: AA: arachidonic acid, DHA: docosahexaenoic acid, AA+DHA: enteral supplementation with AA and DHA in a ratio of 2:1, CI: confidence interval, GA: gestational age, ROP: retinopathy of prematurity, SD: standard deviation, SDS: standard deviation score.

P values were calculated by the Mann–Whitney U test for continuous data, Fisher's exact test to compare two groups, the Mantel–Haenszel chi-square test to compare ordered categorical variables (ROP severity groups) and the Pearson chi-square test for nonordered categorical variables (center).

eTable 3. NfL concentrations (pg/mL) between the AA+DHA-supplemented and standard nutrition groups were compared

	Unadjusted EM (95% CI)			Adjusted EM (95% CI)		
**Variable**	**Ln NfL day 14**	**Ln NfL day 28**	**Ln Mean NfL/day 1st month***	**Ln NfL day 14**	**Ln NfL day 28**	**Ln Mean NfL/day 1st month***
**Standard nutrition**	3.83 (3.64-4.02), n=65	3.43 (3.22-3.63), n=79	4.06 (3.92-4.19), n=86	3.85 (3.67-4.02), n=65	3.42 (3.24-3.60), n=79	4.05 (3.93-4.17), n=86
**AA+DHA**	3.89 (3.68-4.10), n=54	3.22 (3.01-3.44), n=70	3.92 (3.77-4.06), n=77	3.90 (3.71-4.10), n=54	3.28 (3.09-3.48), n=70	3.95 (3.82-4.08), n=77
**p value**	0.684	0.184	0.178	0.676	0.299	0.244

Linear regression was used with the natural logarithm (Ln) of the NfL concentration as the dependent variable.

Model adjusted for gestational age at birth and study center. Estimated means (EM) are reported with 95% CIs.

Abbreviations: AA: arachidonic acid, DHA: docosahexaenoic acid, CI: confidence interval, EM: estimated means, Ln: Natural logarithm, PN: postnatal, NfL: neurofilament light chain

*Calculated as the mean of the area under the curve (AUC) of the concentrations during the first month of life

eTable 4 Relative risk (95% confidence interval) per IVH grade

		Unadjusted RR (95% CI)			Adjusted RR (95% CI)		
**Compared against**	**Variable**	**Ln NfL day 14 (No-IVH n=66)**	**Ln NfL day 28 (No-IVH n=86)**	**Ln Mean NfL/day 1st month* (No-IVH n=99)**	**Ln NfL day 14 (No-IVH n=66)**	**Ln NfL day 28 (No-IVH n=86)**	**Ln Mean NfL/day 1st month* (No-IVH n=99)**
No-IVH,	IVH1	0.97 (0.67-1.42) p=0.873, n=16	1.03 (0.66-1.61), p=0.899, n=20	1.22 (0.92-1.61), p=0.174, n=21	0.86 (0.60-1.24), p=0.412, n=16	0.87 (0.58-1.31), p=0.505, n=20	1.08 (0.84-1.42), p=0.523, n=21
	IVH2	**1.96 (1.40-2.76), p=0.001, n=21**	**1.55 (1.02-2.37), p=0.040, n=23**	**1.81 (1.38-2.38), p<0.001, n=23**	**1.63 (1.16-2.30), p=0.005, n=21**	1.25 (0.85-1.84), p=0.259, n=23	**1.51 (1.16-1.95), p=0.002, n=23**
	IVH3	1.50 (0.90-2.48), p=0.118, N=8	1.57 (0.81-3.04), p=0.182, n=8	**1.54 (1.02-2.32), p=0.039, n=9**	1.24 (0.76-2.04), p=0.390, n=8	1.17 (0.64-2.15), p=0.601, n=8	1.27 (0.86-1.86), p=0.228, n=9
	IVH4	**3.54 (2.13-5.87), p<0.001, n=8**	1.68 (0.97-2.91), p=0.066, n=12	**2.20 (1.51-3.19), p<0.001, n=11**	**3.36 (2.03-5.56), p<0.001, n=8**	1.31 (0.79-2.17), p=0.297, n=12	**1.84 (1.29-2.61), p<0.001, n=11**
No-IVH/IVH1	IVH2-4	**2.11 (1.61-2.78), p<0.001, n=37**	**1.58 (1.15-2.18), p=0.005, n=43**	**1.78 (1.44-2.19), p<0.001, n=43**	**1.85 (1.40-2.46), p<0.001, n=37**	1.28 (0.95-1.74), p=0.097, n=43	**1.50 (1.22-1.84), p<0.001, n=43**

Models adjusted for the intervention group, gestational age at birth, and study center

Linear regression was used with the natural logarithm (Ln) of the NfL concentration as the dependent variable.

Abbreviations: CI: confidence interval, RR: relative risk, NfL: neurofilament light chain, IVH: intraventricular haemorrhage

*Calculated as the mean of the area under the curve (AUC) of the concentrations during the first month of life

eTable 5. Ln NfL concentrations (pg/mL) according to ROP severity and severe ROP

		Unadjusted			Adjusted		
**Independent variable**	**Model and outcome**	**OR (95% CI)**	**p-value**	**Prop. Odds p value**	**OR (95% CI)**	**p-value**	**Prop. Odds p value**
**Ln NfL day 14**	Ordinal regression- ROP severity	1.73 (1.11-2.71)	**0.017**	0.135	1.09 (0.60-1.99)	0.778	0.878
**Ln NfL day 28**		1.76 (1.24-2.49)	**0.001**	0.979	0.98 (0.65-1.49)	0.928	0.150
**Ln Mean NfL/day 1st month***		2.57 (1.59-4.14)	**<0.001**	0.695	1.22 (0.68-2.19)	0.499	0.060
**Ln NfL day 14**	Binary regression- severe ROP	2.05 (1.20-3.49), severe ROP n=35	**0.008**	NA	1.72 (0.79-3.75)	0.170	NA
**Ln NfL day 28**		1.84 (1.23-2.74)	**0.003**	NA	1.14 (0.70-1.87)	0.591	NA
**Ln Mean NfL/day 1st month***		2.71 (1.52-4.85),	**<0.001**	NA	1.47 (0.72-2.98)	0.291	NA

Models adjusted for the intervention group, gestational age at birth, severe IVH (no IVH/ IVH2-4), and study center

Day 14: No ROP n=45, Mild/moderate ROP n=38, Severe ROP n=35, Day 28 No ROP n=60, Mild/moderate ROP n=45, Severe ROP n=43, Mean NfL/day 1sr month: No ROP n=64, Mild/moderate ROP n=51, Severe ROP n=47

Abbreviations: CI: confidence interval, ROP: retinopathy of prematurity, IVH: intraventricular haemorrhage, NfL: neurofilament light chain

*Calculated as the mean of the area under the curve (AUC) of the concentrations during the first month of life

eTable 6. Ln NfL concentrations (pg/mL) according to severe IVH (IVH2-4) and gestational age group (<25/≥25 weeks)

**Gestational age group**	**22-24 weeks**		**25-27 weeks**	
	Unadjusted RR (95% CI)	Adjusted RR (95% CI)	Unadjusted RR (95% CI)	Adjusted RR (95% CI)
Ln NfL day 14	**1.67 (1.04-2.65), p=0.034, IVH2-4 n=20 No IVH, IVH1 n=18**	1.52 (0.87-2.64), p=0.133, IVH2-4 n=20 No IVH, IVH1 n=18	**1.86 (1.33-2.63), p<0.001, IVH2-4 n=17 No IVH, IVH1 n=64**	**1.84 (1.29-2.64), p=0.001, IVH2-4 n=17 No IVH, IVH1 n=64**
Ln NfL day 28	0.81 (0.52-1.25), p=0.331, IVH2-4 n=21 No IVH, IVH1 n=27	0.73 (0.47-1.12), p=0.147, IVH2-4 n=21 No IVH, IVH1 n=27	**1.88 (1.26-2.82), p=0.003, IVH2-4 n=22 No IVH, IVH1 n=79**	**1.84 (1.24-2.72), p=0.003, IVH2-4 n=22 No IVH, IVH1 n=79**
Ln Mean NfL/day 1st month*	1.23 (0.94-1.63), p=0.132, IVH2-4 n=24 No IVH, IVH1 n=30	1.19 (0.89-1.60), p=0.237, IVH2-4 n=24 No IVH, IVH1 n=30	**1.82 (1.37-2.41), p<0.001, IVH2-4 n=19 No IVH, IVH1 n=90**	**1.74 (1.32-2.29), p<0.001, IVH2-4 n=19 No IVH, IVH1 n=90**

Linear regression was used with the natural logarithm (Ln) of the NfL concentration as the dependent variable.

Model adjusted for gestational age at birth, study center, and intervention group

Abbreviations: CI: confidence interval, Ln: Natural logarithm, NfL: neurofilament light chain, ROP: retinopathy of prematurity

*Calculated as the mean of the area under the curve (AUC) of the concentrations during the first month of life

eTable 7. Ln NfL concentrations (pg/mL) according to severe ROP and gestational age group (<25/≥25 weeks GA)

	<25 weeks GA		≥25 weeks GA	
**Gestational age group**	**Unadjusted OR (95% CI)**	**Adjusted OR (95% CI)**	**Unadjusted OR (95% CI)**	**Adjusted OR (95% CI)**
Ln NfL day 14	0.99 (0.41-2.40), p=0.987,<br>Severe ROP n=23<br>No/mild/moderate ROP n=15	1.60 (0.51-5.03), p=0.418<br>Severe ROP n=23<br>No/mild/moderate ROP n=15	1.49 (0.63-3.52), p=0.359,<br>Severe ROP n=12<br>No/mild/moderate ROP n=68	2.26 (0.81-6.29) p=0.120<br>Severe ROP n=12<br>No/mild/moderate ROP n=68
Ln NfL day 28	0.83 (0.39-1.80), p=0.641<br>Severe ROP n=26<br>No/mild/moderate ROP n=22	1.17 (0.48-2.86), p=0.738<br>Severe ROP n=26<br>No/mild/moderate ROP n=22	**1.76 (1.03-3.02), p=0.038,<br>Severe ROP n=17<br>No/mild/moderate ROP n=83**	**1.92 (1.05-3.50), p=0.033<br>Severe ROP n=17<br>No/mild/moderate ROP n=83**
Ln Mean NfL/day 1st month*	0.96 (0.33-2.76), p=0.933,<br>Severe ROP n=29<br>No/mild/moderate ROP n=25	0.89 (0.27-2.89), p=0.846,<br>Severe ROP n=29<br>No/mild/moderate ROP n=25	**2.35 (1.03-5.38), p=0.043,<br>Severe ROP n=18<br>No/mild/moderate ROP n=90**	**2.62 (1.06-6.51), p=0.038,<br>Severe ROP n=18<br>No/mild/moderate ROP n=90**

Binary regression with severe ROP as dependent variable

Models adjusted for intervention group and severe IVH (no IVH/ IVH2-4),

Abbreviations: CI: confidence interval, Ln: Natural logarithm, NfL: neurofilament light chain, ROP: retinopathy of prematurity

*Calculated as the mean of the area under the curve (AUC) of the concentrations during the first month of life
